# Imaging of Peripheral Intraneural Tumors: A Comprehensive Review for Radiologists

**DOI:** 10.3390/cancers17020246

**Published:** 2025-01-13

**Authors:** Kapil Shirodkar, Mohsin Hussein, Pellakuru Saavi Reddy, Ankit B. Shah, Sameer Raniga, Devpriyo Pal, Karthikeyan P. Iyengar, Rajesh Botchu

**Affiliations:** 1 Royal Orthopaedic Hospital, Birmingham B31 2AP, UK; 2 Eclat Imaging Centre, Mumbai 400056, India; 3 Sultan Qaboos University Hospital, Seeb H5QC+4HX, Oman; 4 Stoke Mandeville Hospital, Aylesbury HP21 8AL, UK; 5 Southport and Ormskirk Hospital NHS Trust, Southport PR8 6PN, UK

**Keywords:** multiparametric magnetic resonance imaging, magnetic resonance imaging biopsy, neoplasms, radiologists

## Abstract

Background/Objectives: Intraneural tumors (INTs) pose a diagnostic challenge, owing to their varied origins within nerve fascicles and their wide spectrum, which includes both benign and malignant forms. Accurate diagnosis and management of these tumors depends upon the skills of the radiologist in identifying key imaging features and correlating them with the patient’s clinical symptoms and examination findings. Methods: This comprehensive review systematically analyzes the various imaging features in the diagnosis of intraneural tumors, ranging from basic MR to advanced MR imaging techniques such as MR neurography (MRN), diffusion tensor imaging (DTI), and dynamic contrast-enhanced (DCE) MRI. Results: The article emphasizes the differentiation of benign from malignant lesions using characteristic MRI features, such as the “target sign” and “split-fat sign” for tumor characterization. The role of advanced multiparametric MRI in improving biopsy planning, guiding surgical mapping, and enhancing post-treatment monitoring is also highlighted. The review also underlines the importance of common diagnostic pitfalls and highlights the need for a multi-disciplinary approach to achieve an accurate diagnosis, appropriate treatment strategy, and post-therapy surveillance planning. Conclusions: In this review, we illustrate the main imaging findings of intraneural tumors, focusing on specific MR imaging features that are crucial for an accurate diagnosis and the differentiation between benign and malignant lesions.

## Simple Summary

Intraneural tumours are difficult to diagnose. A plethora of pathologies can involve the nerve from schwannoma, neurofibroma, intraneural ganglion to more esoteric intraneural tumours like ewings sarcoma, metastasis, synovial sarcoma and perineuroma. The imaging features of these can be variable and management of intraneural lesions requires a mutidisciplinary approach to decrease morbidity and mortality. This paper aims to describe the key MRI characteristics which can help differentiate between the benign and malignant tumours, aid in biopsy and surgical planning as well as help in post-treatment monitoring.

## 1. Introduction

This article provides a detailed review of the imaging methods used to evaluate intraneural tumors (INTs). It describes the gold standard role of magnetic resonance imaging (MRI) and touches on advanced MR imaging protocols, highlighting the key MR imaging descriptors necessary in radiology reports to accurately identify and characterize intraneural tumors. The article showcases a wide range of intraneural tumors, including primary neurogenic and metastatic tumors, in a pictorial format, with a discussion of the radiological signs used to determine if a lesion is intraneural or extraneural, as well as benign or malignant. It also explores the roles of different members of the MDT team in the management and decision-making regarding intraneural tumors. This is supported by a review of the relevant and up to date literature on imaging findings, guidelines, and recommendations [1].

**Figure 1:**
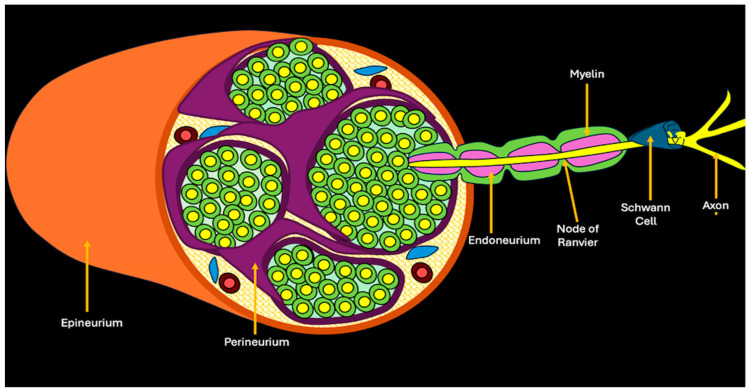
Illustration showing the normal nerve micro-architecture, depicting the various nerve layers and cellular units.

Intraneural tumors (INTs) are a heterogeneous group of neoplasms involving one or more elements within peripheral nerves. A nerve consists of many fascicles, each with multiple axons surrounded by three layers of connective tissue: the endoneurium (an inner layer surrounding individual axons), the perineurium (a multilayered membranous sheath that surrounds the fascicles), and the epineurium (the outermost connective tissue layer) [2] (Figure 1).

INTs represent 12% of all benign soft tissue tumors and approximately 8% of malignant soft tissue varieties, with a combined incidence rate close to approximately 5% [2,3,4]. These can be either benign (e.g., schwannomas, neurofibromas, and perineuromas) primary malignant nerve sheath tumors or secondary malignancies invading the nerves (metastases, lymphomas, and sarcomas). Benign tumors such as schwannomas and neurofibromas are the most common intraneural tumor masses [5,6,7] and together constitute the vast majority of benign intraneural tumors [8].

## 2. Imaging Techniques and Their Appropriateness

The selection of imaging technique should align with the clinical inquiry and potential differential diagnosis, considering the tumor’s intraneural location, anticipated histopathology, aggressiveness, and feasibility of surgical planning.

### 2.1. Ultrasound (US)

Ultrasound serves as the primary tool for assessing superficial soft tissue lesions, including peripheral nerve tumors, with a focus on lesion identification, characterization of solid versus cystic nature, intratumoral vascularity, and necrosis [9]. Its high axial resolution and dynamic capabilities provide clear visualization of the tumor–nerve relationship, confirming continuity with the parent nerve [10]. This modality is especially beneficial for benign peripheral nerve sheath tumors, notably in pediatric patients with neurofibromatosis, who often undergo frequent and long-term imaging assessments and follow-ups [11,12]. Benign tumors typically appear as well-defined, hypoechoic masses on high-resolution ultrasound (HRUS) via high-frequency linear transducers [13]. Schwannomas usually present as eccentric masses in relation to the parent nerve, whereas neurofibromas are generally located centrally within the parent nerve [10]. Furthermore, ultrasound facilitates real-time imaging, biopsy guidance, and vascularity assessments [13,14], while presenting advantages such as portability, cost-effectiveness, and improved patient comfort compared with MR (claustrophobia and sound concerns) and CT (radiation concerns). Ultrasound examinations also provide opportunities for direct patient interaction and immediate verbal feedback, thereby enhancing the diagnostic confidence of imaging [15].

Intraoperative high-resolution ultrasound (iHRU) enhances surgical planning by accurately visualizing tumor morphology and nerve structures, facilitating effective surgical resection planning [16]. Additionally, it helps to distinguish schwannomas (which appear as smooth circumscribed masses with hypoechoic enlarged fascicles) from malignant peripheral nerve sheath tumors (MPNSTs) (which appear as irregularly shaped masses with variable heterogeneous intralesional echotextures) [16]. iHRU also allows for better intraoperative identification of nerve fascicles and thus helps reduce the risk of iatrogenic nerve injury [17].

Contrast-enhanced ultrasound (CEUS) may provide vital information about intraneural tumor vascularity and perfusion, aiding in the differentiation of benign from malignant lesions [16,18,19,20].

**Figure 2:**
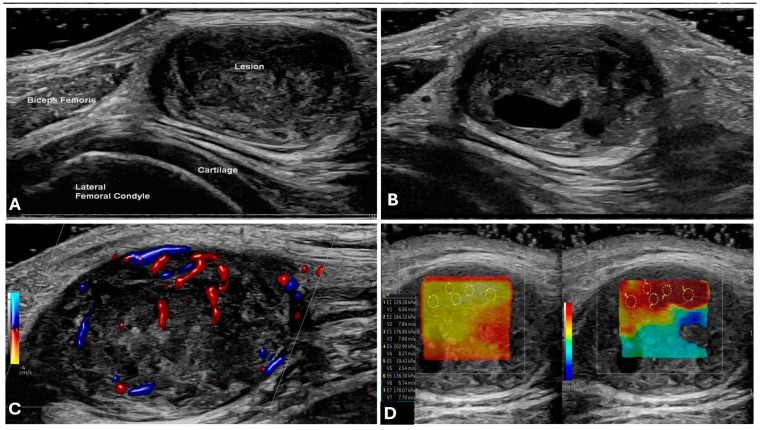
Benign peripheral nerve sheath tumor (schwannoma) of the tibial nerve in a 42 year old woman. Sagittal greyscale ultrasound (**A**,**B**), sagittal color Doppler (**C**), and sagittal elastography (**D**) images showing a well-circumscribed, heterogenous echogenicity lesion (arrows) with mild organized intrinsic vascularity (thick arrows).

Emerging technologies include ultrasound elastography (UE) (which allows the independent measurement of tissue stiffness within intraneural tumors) and ultrahigh-frequency ultrasound transducers (up to 70 MHz) (which can visualize internal neuronal architecture) [7,8,9]. However, owing to limitations such as a restricted field of view and the dependence on operator skill, ultrasound often serves as a supplementary tool rather than a primary imaging modality such as MRI (Figure 2) [20,21]

### 2.2. Computed Tomography (CT)

The utility of CT in the primary diagnosis of intraneural tumors is limited [8]. However, it excels in assessing bone involvement, distinguishing smooth bony remodeling (due to benign INTs) from cortical erosion and periosteal reactions (associated with malignant lesions). CT is also useful for identifying intralesional calcification and performing systemic staging for malignant tumors. It is also useful for surgical biopsy planning, presurgical three-dimensional tumor mapping and determining angiographic roadmaps during preoperative embolization of hypervascular tumors [22,23].

### 2.3. Magnetic Resonance Imaging (MRI)

Magnetic resonance imaging (MRI) is regarded as the definitive gold standard and the preferred imaging modality for the assessment of peripheral nerve sheath tumors (PNSTs), especially for presurgical planning of deep-seated soft tissue lesions or overweight patients with high body mass indices (BMIs) [24]. MRI offers exceptional soft tissue contrast across multiple imaging planes and is more effective in characterizing the tumor morphology, as well as delineating the extent of the tumor, particularly in the context of plexiform neurofibromas or tumors that are in anatomically complex regions such as the spine and retroperitoneum.

The conventional MRI protocol dedicated to the imaging of peripheral nerve sheath tumors (PNSTs) includes T1-weighted (T1-w), T2-weighted (T2-w), and fat-saturated or inversion recovery fluid-sensitive sequences (such as T2-weighted fat-suppressed (T2-FS) or short tau inversion recovery (STIR) sequences), generally followed by postcontrast T1-w fat-suppressed sequences (Gd+ PC T1-w FS) [25] (F 1) (Table 1). It is likely that imaging sequences will have institutional variation but a combination of fluid-sensitive, anatomic (T1) sequences and imaging in at least three planes is required.

T1-weighted sequences offer exceptional soft tissue clarity, facilitating comprehensive assessment of intraneural tumor characteristics, including margins and spatial relationships with adjacent tissues. These sequences also reveal fatty components and the split-fat sign in nerve sheath tumors [26], while aiding in the identification of subacute hemorrhage in schwannomas and MPNSTs. Melanin appears hyperintense in T1W images and is observed in malignant melanotic nerve sheath tumors (MMNSTs) [27].

T2-weighted sequences highlight the internal signal intensity of PNSTs and assist in evaluating peritumoral edema [24], enhancing diagnostic confidence through identifiable patterns [24,28].

Fluid-sensitive fat-suppressed sequences (T2-weighted FS or STIR) improve lesion visibility and detect subtle peritumoral edema (PTE), making them crucial for identifying small lesions [29].

Gadolinium-enhanced T1-weighted sequences may be useful for evaluating tumor vascularity and the internal architecture distinguishing tumoral enhancement patterns, particularly in the context of heterogeneous enhancement in schwannomas compared with neurofibromas [30] and detecting malignant transformation in neurofibromas [31].

DWI/ADC imaging aids in characterizing tumor composition and cellularity, offering a reliable method for differentiating between benign and malignant tumors [32,33,34].

Advanced MR techniques are described below. It is recognized that these techniques are not widely available in low- and middle-income countries and, even where they are available, there is variability in their adoption and perceived utility.

Diffusion tensor imaging (DTI) and tractography enables visualization of the integrity of peripheral nerves and delineates the spatial relationship between the tumor and nerve fibes (how close the tumor is to the parent nerve fibers) [35]. It facilitates the evaluation of peripheral nerve integrity and the spatial relationship between tumors and nerve fibers, aiding in presurgical planning to protect vital nerve tracts. Furthermore, it helps to distinguish between schwannomas (displaced nerve fibers), neurofibromas (splayed nerve fibers), and MPNSTs (infiltrated and destroyed nerve fibers). This is of great importance during surgical resection, as it helps in preserving healthy nerves during surgical procedures or limits the involvement of affected nerves [25,36,37]. However, its adoption is limited by prolonged acquisition times, artefacts at nerve fiber crossings, and the complexity of image interpretation.

MR neurography (MRN) combines advanced imaging techniques to yield high-resolution images of peripheral nerves, offering insights into nerve morphology and tumor relationships [38]. It is particularly useful for assessing the tumoral involvement of nerve fascicles and detecting subtle abnormalities that are not routinely visible on conventional MRI [8,29,38]. Despite its benefits, MRN faces challenges related to image acquisition and interpretation that hinder broader use.

Dynamic contrast-enhanced (DCE) MRI involves rapid imaging sequences acquired before, during, and following the injection of a gadolinium-based contrast agent and provides valuable insights into tumor vascularity, perfusion, and permeability [39]. It distinguishes benign from malignant PNSTs based on various enhancement patterns (type 1–3 enhancement curves) [33], aiding in biopsy targeting and treatment monitoring. DCE-MRI can be used to identify areas of increased vascularity for biopsy targeting and monitoring treatment responses, particularly with antiangiogenic therapies [40]. Nonetheless, the variability in DCE acquisition protocols and complex quantitative analysis limits its universal application.

Although not routinely utilized, MR spectroscopy (MRS) offers metabolic insights into PNSTs [41], with elevated choline peaks potentially indicating malignancy. This method faces technical challenges [42] in obtaining quality spectra from peripheral lesions, leading to limited spatial resolution. Advanced imaging techniques, when combined with conventional MRI, enhance diagnostic accuracy and surgical planning for intraneural tumors, but MRS requires specialized expertise for interpretation.

### 2.4. PET-CT

PET/CT is essential for diagnosing intraneural tumors, providing both anatomical and metabolic insights. Malignant tumors typically show elevated FDG uptake, whereas benign tumors present lower levels, aiding in NF-1 lesion characterization [43]. PET/CT also has a variable role in staging the tumor and planning therapy, treatment planning, and monitoring the response by measuring the changes in FDG uptake [44,45]. As benign conditions such as inflammation or metabolically active benign tumors can also exhibit FDG uptake, interpretation alongside the wider clinical context is required [46].

## 3. MRI-Based Approach to an Intraneural Tumor

### 3.1. When to Suspect a Neurogenic Tumor on MRI

**Figure 3:**
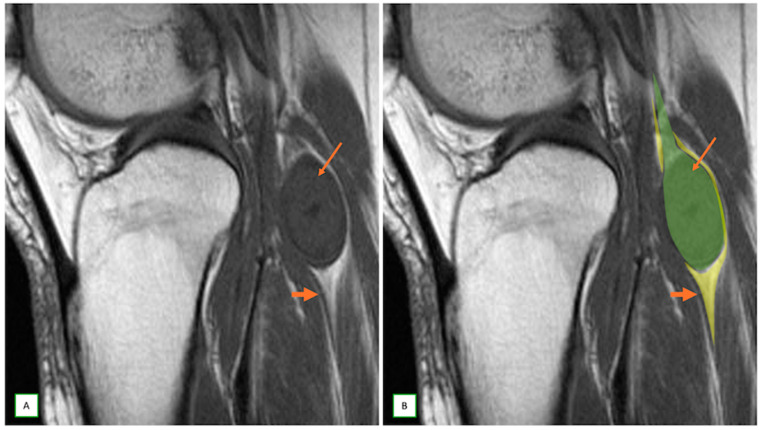
Benign nerve sheath tumor (schwannoma) in a 48-year-old man with a popliteal fossa lump. Sagittal T1 (**A**) and Sagittal T2 overlay illustration (**B**) images with characteristic “split-fat sign” (thick arrow) and shows the “tail-sign” with the nerve entering and exiting the tumor (thin arrows).

**Figure 4:**
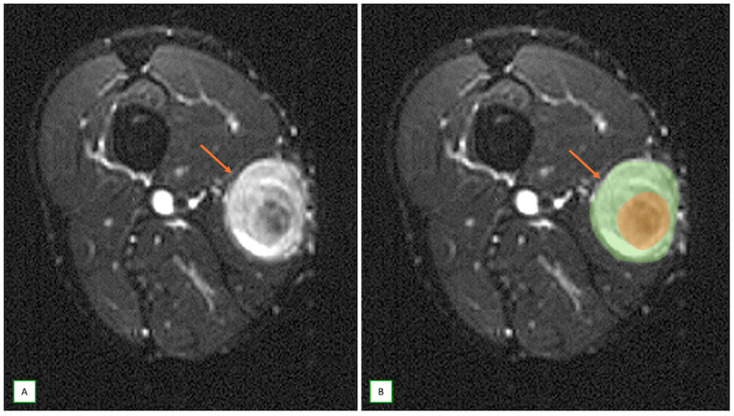
Benign nerve sheath tumor (neurofibroma) in a 65-year-old man with a lump in the thigh. Axial fat-saturated T2 axial (**A**) and axial fat-saturated T2 overlay illustration (**B**) images showing the characteristic “target sign” (thin arrows).

**Figure 5:**
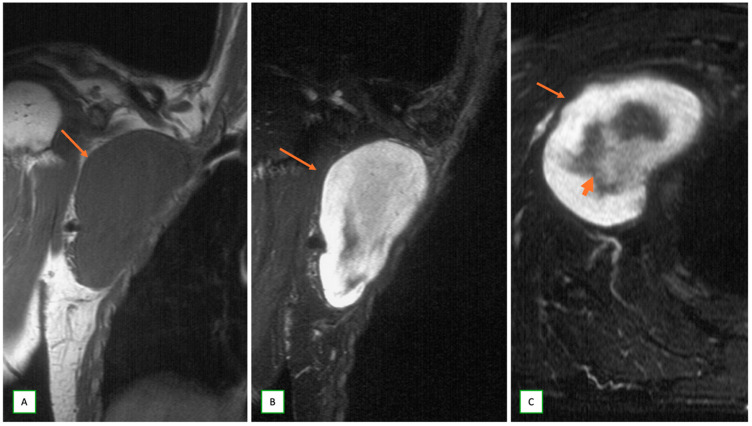
Benign neurofibroma in a 67-year old man presenting with right arm pain. Coronal T1 (**A**), coronal (**B**) and axial (**C**) fat saturated T2 images showing a lesion of the brachial plexus with a well-circumscribed cylindrical or ovoid shape (thin arrows). It has low to intermediate T1 signal, high T2 signal, and shows “target sign” on T2-w sequences (thick arrow).

**Figure 6:**
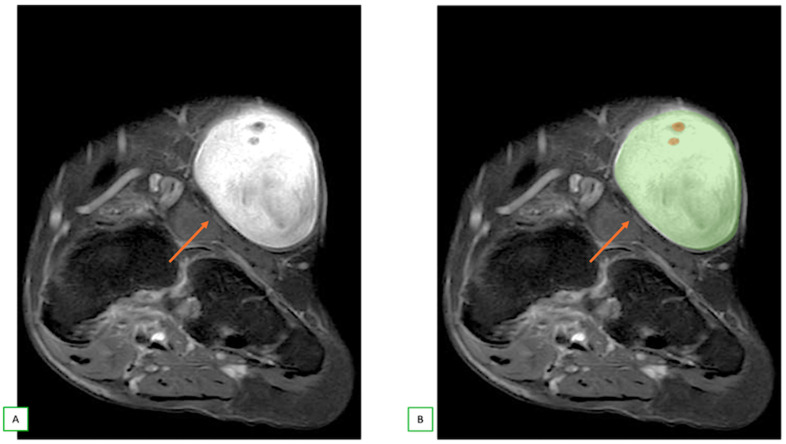
Benign nerve sheath tumor (neurofibroma) in a 61-year-old man with a swelling of the foot. Coronal fat-saturated T2 (**A**) and coronal fat-saturated T2 with color overlay (**B**) illustration images with characteristic “fascicular sign”.

When MRI studies are performed, several key features can lead to suspicion of a neurogenic tumor. The most important sign is direct continuity with a neural structure or location along a typical nerve distribution [8]. Neurogenic tumors appear as fusiform or oval lesions with clear, well-defined margins. They often exhibit the “split-fat sign”, characterized by a thin rim of fat encircling around the lesion on T1-weighted images, suggesting an intermuscular origin [47,48] (Figure 3). The presence of the “target sign” on T2-weighted images is highly suggestive of a peripheral nerve sheath tumor (such as a schwannoma or neurofibroma), marked by central T2-weighted hypointensity surrounded by peripheral hyperintensity (Figure 4 and Figure 5) [30]. Another characteristic finding is the “fascicular sign”, which manifests as numerous small ring-like structures on T2-weighted images, indicating enlarged fascicles surrounded by the perineurium [25] (Figure 6). The “tail sign” is pathognomonic for peripheral nerve sheath tumors and is caused by the parent nerve entering and exiting the tumor [49].

Neurogenic tumors typically exhibit low to intermediate signals on T1-weighted MR images and high signals on T2-weighted images, as well as variable enhancement characteristics following gadolinium contrast administration [29]. The presence of complex, well-circumscribed masses along the anticipated or expected course of major nerves or plexuses, particularly in deeper anatomical locations, such as the retroperitoneum or mediastinum, suggests a possible neurogenic origin. Other soft tissue sarcomas may have an irregular growth pattern [47]. Denervation or fatty atrophy of the target musculature may be present in chronic intraneural tumors but is rarely found in other soft tissue tumors [50]. Finally, multiple soft tissue lesions in a young patient with bilateral vestibular schwannomas or multiple (>6) café-au-lait stigmata may raise the issue of genetic syndromes such as neurofibromatosis or schwannomatosis [24].

### 3.2. Differentiating Neurofibroma from Schwannoma on MRI

**Figure 7:**
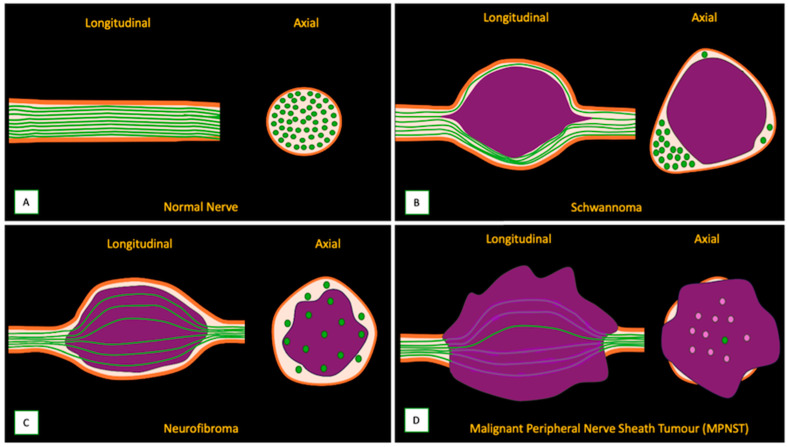
Illustrations showing a comparison of imaging features of normal and pathological nerves. (**A**) Longitudinal and axial images of the normal nerve, (**B**) eccentric growth pattern of schwannoma displacing the residual nerve fibers to the periphery, (**C**) neurofibroma showing a typical growth pattern splaying fascicles around the tumor, (**D**) illustration showing a malignant peripheral nerve sheath tumor (MPNST) with an infiltrative growth pattern disrupting the fascicular architecture and extra-neural extension.

**Figure 8:**
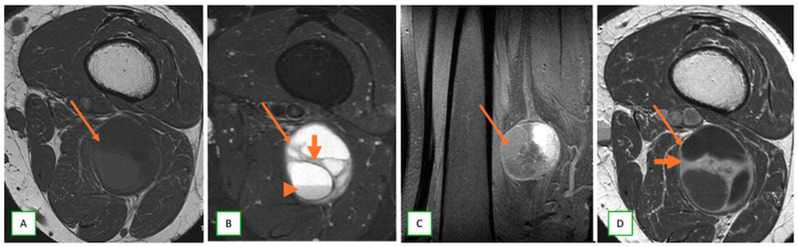
Schwannoma of the sciatic nerve in a 61-year-old man with right thigh pain. Axial T1 (**A**), axial (**B**), sagittal (**C**) fat-saturated T2, and axial T2 axial (**D**) images showing cystic degeneration, multiple internal septations (thick arrows), and fluid–fluid levels (arrowheads) representing blood products of differing age.

**Figure 9:**
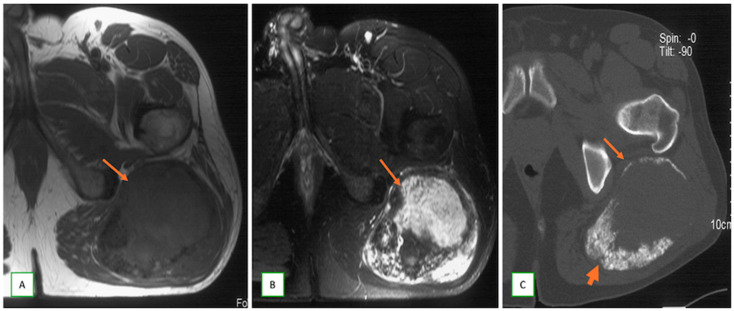
Malignant peripheral nerve sheath tumor (MPNST) of the sciatic nerve in a 51-year old man presenting with left buttock swelling. Axial T1 (**A**), axial fat saturated T2 (**B**), and axial CT (**C**) images showing the large, heterogeneous mass (thin arrows) with low to intermediate T1 signal, high T2 signal with central necrosis/hemorrhage, and peripheral calcification (thick arrows) on CT.

Several essential MR imaging features can help distinguish between neurofibroma and schwannoma. Schwannomas are typically eccentric to the nerve, displacing nerve fibers to the periphery. Neurofibromas are usually central within the nerve, often enlarging it diffusely. MPNSTs tend to be more central, irregular, and lobulated and may show infiltrative borders and disruption of the fascicular architecture [30,51] (Figure 7). While both can exhibit a target sign on T2-weighted images, it is reported to be more common and well defined in schwannomas. Neurofibromas may show a less distinct target appearance [47]. Both schwannomas and neurofibromas often demonstrate a fascicular sign, but this has been reported to have marked variation in retrospective studies [25,47]. In schwannomas, the entering and exiting nerves are often visible at the poles of the tumor, giving rise to the tail sign. In neurofibromas, the nerve entry and exit points are less distinct because of the central position of the tumor within the nerve [8]. Schwannomas show more heterogeneous enhancement, often with a peripheral pattern. Neurofibromas typically demonstrate more homogeneous or central enhancement [33]. Cystic degeneration and hemorrhagic changes are seen far more frequently in schwannomas than in neurofibromas (Figure 8). These changes are more common in large schwannomas than in their smaller counterparts [26]. MPNSTs (Figure 9) tend to show more extensive areas of cystic degeneration, hemorrhage, or necrosis.

**Figure 10:**
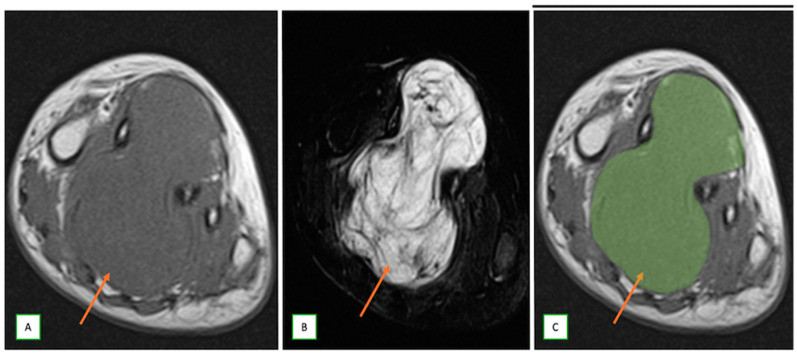
Neurofibroma of the foot in a 58-year old woman presenting with a swollen right foot. Coronal T1 (**A**), coronal fat saturated T2 (**B**), and coronal T1 coronal overlay illustration (**C**) images. A neurofibroma (thin arrows) extending from the plantar to the dorsal foot compartments via the second intermetatarsal web space giving rise to the characteristic “dumb-bell” shape with a “waist” or narrowing at the level of the second intermetatarsal web space.

**Figure 11:**
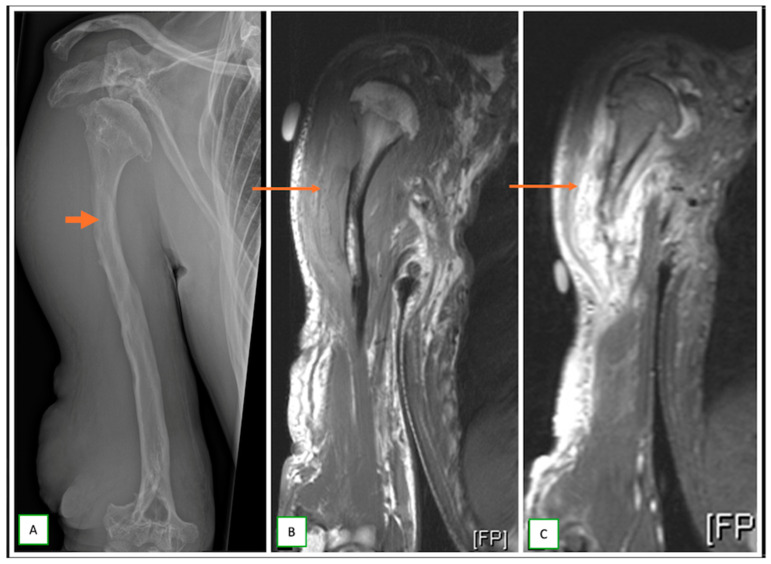
Plexiform neurofibroma in a 75-year-old man with a case of neurofibromatosis type 1 who presented with a right arm painful lump. The frontal radiograph of the right humerus (**A**) shows a congenital dysplasia/remodeling with a bowed gracile right humerus (Thick arrows). Coronal T1 (**B**) and coronal fat-saturated T2 (**C**) images show a plexiform neurofibroma (Thin arrows) which appears as a diffusely long segment enlargement of multiple arm nerves akin to a “bag of worms”.

**Figure 12:**
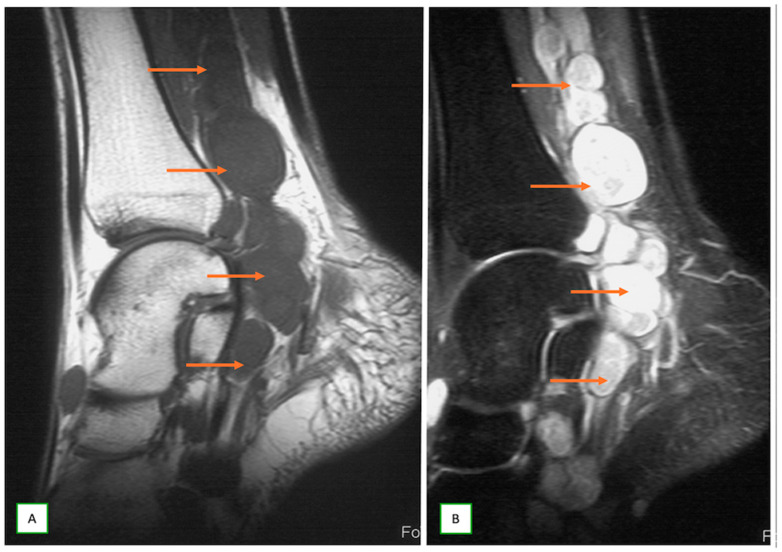
Multiple contiguous nerve sheath tumors of the tibial nerve in a case of schwannomatosis syndrome in a 37-year-old man. Sagittal T1 (**A**) and sagittal fat-saturated T2 (**B**) images show the multiple lesions (thin arrows) that appear low on T1 and high on T2-weighted images.

Paraspinal PNSTs have a more characteristic dumbbell shape, due to an intraspinal and extraspinal component, with an expanded neural canal between the two components through intraspinal extension. The dumb-bell shape can also be observed when a neurofibroma extends across two compartments via a narrow passage (Figure 10). In neurofibromatosis type 1 (NF1) syndrome, the presence of multiple neurofibromas is the hallmark of the syndrome and even forms part of its diagnostic criteria (two or more neurofibromas of any type or one plexiform neurofibroma). The plexiform neurofibroma (Figure 11) has a “bag of worms” due to expansion and enlargement of the nerves. Multiple schwannomas are often observed with syndromic associations, such as schwannomatosis or NF2 syndrome [24] (Figure 12).

Schwannomas display diffusion restrictions on DWI/ADC images with lower apparent diffusion coefficient (ADC) values than neurofibromas. A cut-off ADC value of 1.7 × 10^−3^ mm^2^/s has been suggested to differentiate between the two [36]. On diffusion tensor imaging (DTI), schwannomas tend to displace nerve fibers over their tumor capsule. Neurofibromas typically show splayed and partially disrupted nerve fibers throughout the tumor [25]. Calcifications are more common in schwannomas, especially long-standing tumors or “ancient schwannomas”. Calcifications are rare in neurofibromas [26].

Although the above MR imaging features can reveal some degree of overlap between schwannomas and neurofibromas, a definitive diagnosis ultimately requires histopathological confirmation via image-guided biopsy.

### 3.3. Differentiating Between Benign and Malignant PNSTs

Differentiating between benign and malignant peripheral nerve sheath tumors (PNSTs) based on imaging alone can often be difficult. However, there are few diagnostic clues that may help in the appropriate triage of patients (Table 2).

**Figure 13:**
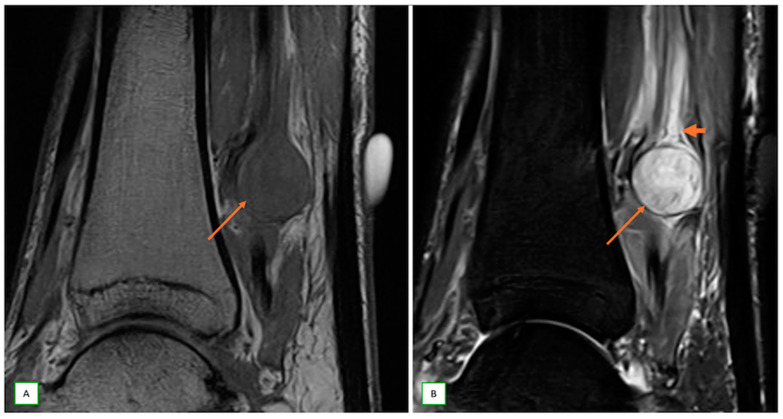
Benign nerve sheath tumor (neurofibroma) in a 47-year-old woman with a lump behind the leg. Sagittal T1 (**A**), fat-saturated T2 (**B**) images showing peri-tumoral edema (thick arrow) around a neurofibroma (thin arrows).

Compared with BPNSTs, MPNSTs are usually larger than 5 cm in diameter and have a rapid growth rate [52]. Unlike the well-circumscribed margins of BPNSTs, MPNSTs often present with poorly defined margins, as they infiltrate adjacent soft tissues [30]. MPNSTs generally show heterogeneous signal intensity on both T1- and T2-weighted images, with central necrosis or hemorrhagic areas, and often lack the characteristic T2-W “target signs” of BPNSTs [8]. MPNSTs typically show early heterogeneous arterial contrast enhancement with rapid washout on dynamic studies [33]. Peritumoral edema (PTE) is more common and extensive in MPNSTs than in BPNSTs, and is minimal and often absent [34] (Figure 13). MPNSTs tend to have lower apparent diffusion coefficient (ADC) values than benign PNSTs, with a suggested cut-off of 1.0–1.1 × 10^−3^ mm^2^/s [53]. Diffusion tensor imaging (DTI) may reveal more extensive disruption of nerve fibers in MPNSTs [25]. MPNSTs commonly show a high FDG uptake, often greater than 3.5 SUVmax, mainly in patients with type 1 neurofibromatosis [43].

As imaging findings alone sometimes cannot be used to confidently distinguish benign from malignant PNSTs, image-guided biopsy is still the standard for definitive diagnosis [54].

### 3.4. When to Suspect Malignant Transformation in a Benign PNST

Detecting potential malignant transformation of a previously benign peripheral nerve sheath tumor (BPNST) is important for enabling timely intervention and complication prevention.

A sudden noticeable enlargement of a BPNST from its otherwise stable appearance, with tumor growth rates of more than 2 cm per year or an increase in volume of 20%, is concerning for malignant transformation [55]. The appearance of a new-onset or sudden worsening of pain can potentially herald ongoing malignant transformation [5]. Acute or subacute rapid interval progression of neurological deficits in the distribution of the affected nerve often results in red flags for underlying malignant transformation [52]. Interval loss of the characteristic “target sign” on T2-weighted MR images [30]; interval infiltration into adjacent structures and the development of irregular margins [31]; interval change from intralesional homogenous signals to heterogeneous signals on both T1- and T2-weighted images [25]; interval appearance of necrotic or hemorrhagic areas within the tumor [8]; rapid interval increase in peritumoral edema [34]; and heterogeneous enhancement [33] are all potential red flags for an underlying malignant transformation. An interval high FDG uptake with an SUVmax > 3.5 on PET/CT is highly suspicious for malignant transformation in NF-1 patients [43]. A significant decrease in the apparent diffusion coefficient (ADC) can suggest that increased cellularity is associated with malignant transformation [53]. While size alone is not a definitive imaging feature, PNSTs larger than 5 cm are more likely to be malignant or undergo malignant transformation [56]. Peripheral nerve sheath tumors that develop in deep and complex anatomical regions, such as the retroperitoneum or mediastinum, present an inherent increased risk of malignant transformation [57]. Individuals with syndromic associations or previous radiation therapy are predisposed to an elevated risk of malignant transformation of their neurofibromas, particularly plexiform neurofibromas [58,59]. In cases where multiple concerning features are present, especially in high-risk patients, prompt evaluation and consideration of biopsy or surgical intervention are warranted.

## 4. Imaging Differential Diagnosis

**Figure 14:**
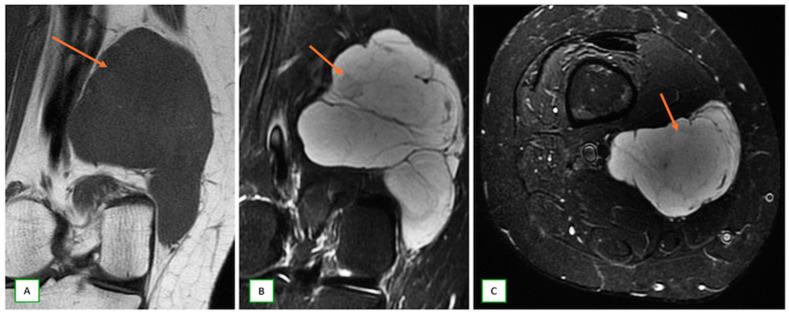
Myxoid nerve sheath tumor in a 59-year-old woman with a lump behind the knee. Coronal T1 (**A**), coronal (**B**), and axial (**C**) fat-saturated T2 images show a myxoid tumor (thin arrows) showing an extremely high signal on the T2-weighted images.

**Figure 15:**
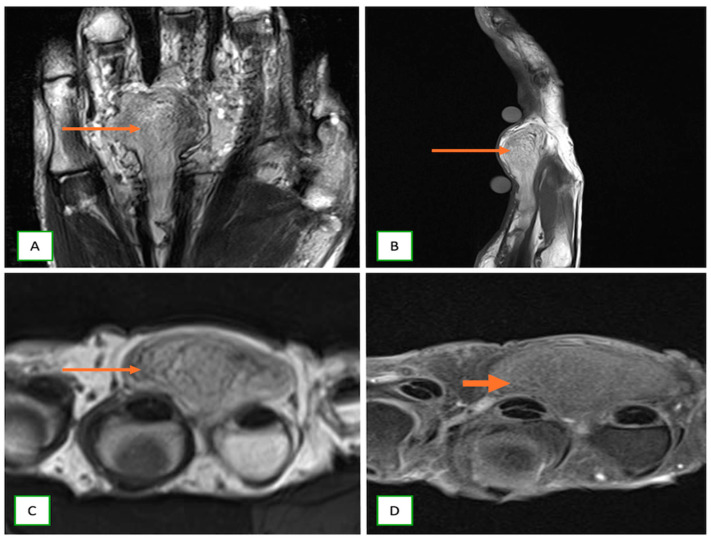
Lipofibromatous hamartoma of a digital nerve in a 53-year-old woman with a lump in their right hand. Coronal T1 (**A**), sagittal T2 (**B**), axial T1 (**C**), and axial fat-saturated T2 (**D**) fat-saturated T2 images showing the fatty components (thin arrows) appearing hyperintense on both T1 and T2-weighted images with suppression of signal on the fat-saturation images (thick arrows).

**Figure 16:**
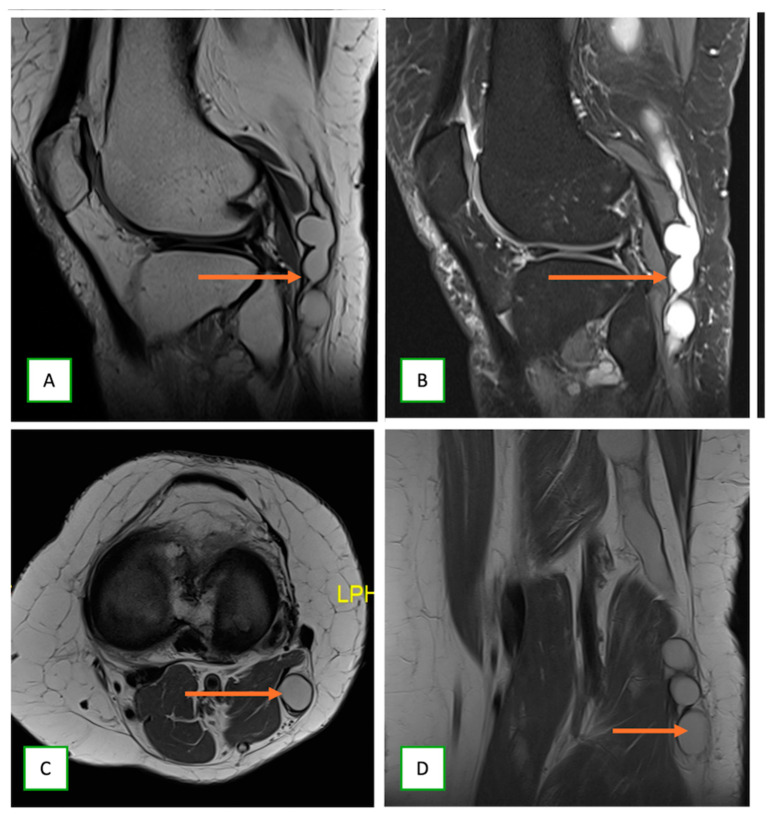
Intraneural ganglion cyst of the tibial nerve in a 39-year-old woman with a painful lump behind the left knee. Sagittal T1 (**A**), sagittal fat-saturated T2 (**B**), axial T1 (**C**), and coronal T1 (**D**) images show the cystic lesion within the nerve (thin arrow), which appears low on T1- and high on T2-weighted images.

**Figure 17:**
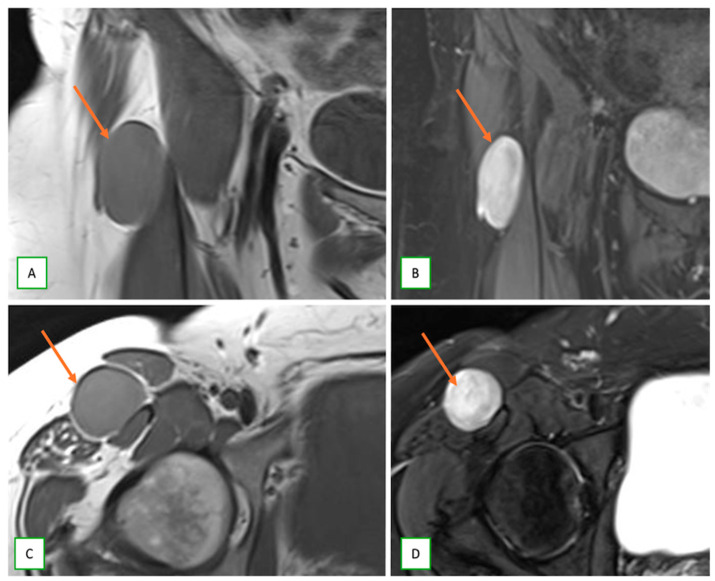
Intraneural perineuromas of the lateral femoral cutaneous nerve in a 49-year-old woman. Coronal T1 (**A**) and fat-saturated T2 (**B**), axial T1 (**C**), and fat-saturated T2 (**D**) images showing fusiform nerve enlargement (thin arrow) with low to intermediate T1 signal and high T2 signal.

**Figure 18:**
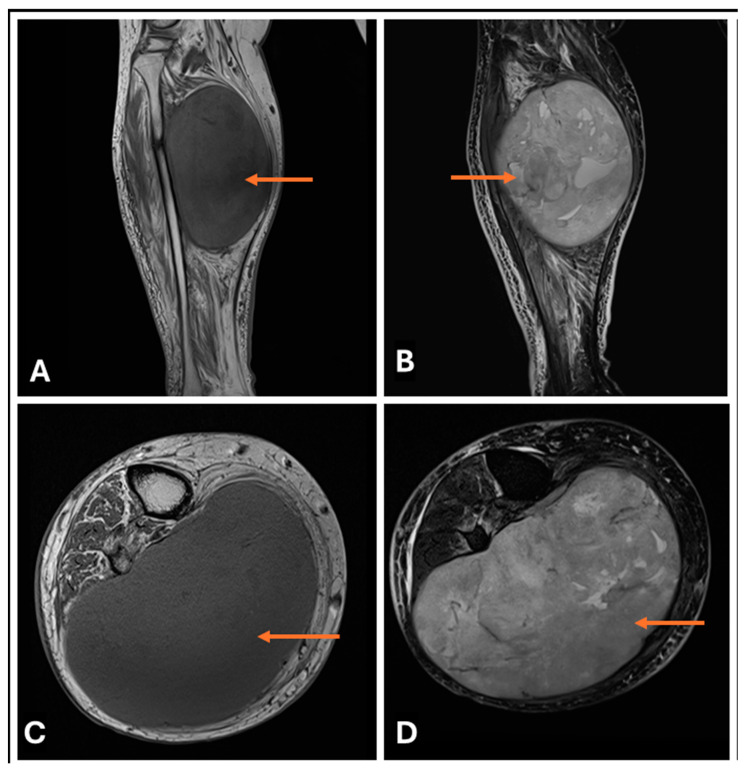
(**A**–**D**) Benign Triton tumour in a 45-year old man presenting with (**A**) T1-w Sagittal; (**B**) T2-w FS Sagittal; (**C**) T1-w Axial and (**D**) T2-w axial images showing Triton Tumour (thin arrows) with Fusiform nerve enlargement and heterogeneous intralesional signal in a benign triton tumor (neuromuscular choristoma).

**Figure 19:**
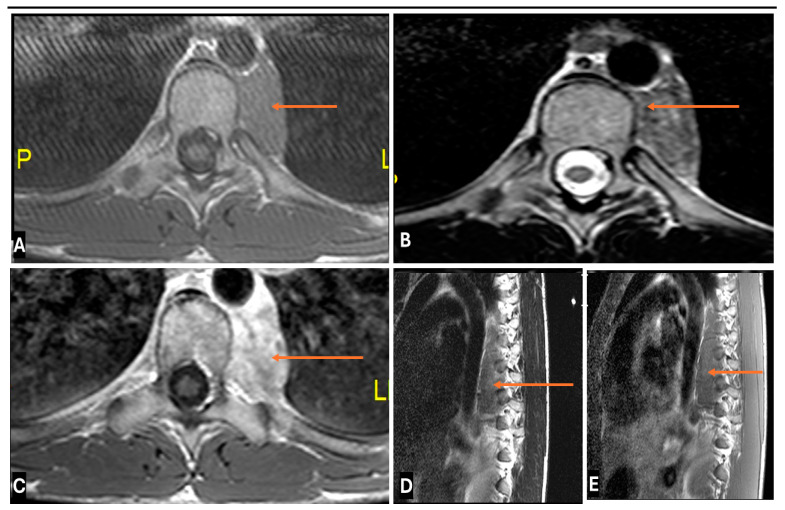
Benign Ganglioneuromas in a 38-year old female. (**A**–**E**). (**A**) T1-w Axial; (**B**) T2-w Axial; (**C**) T1-w FS Post contrast Axial (**D**) T1-w Sagittal (**E**) T2-w FS Sagittal images showing Ganglioneuromas as a paraspinal mass lesion with Low to intermediate T1 signal, heterogeneous high T2 signal and delayed progressive enhancement.

**Figure 20:**
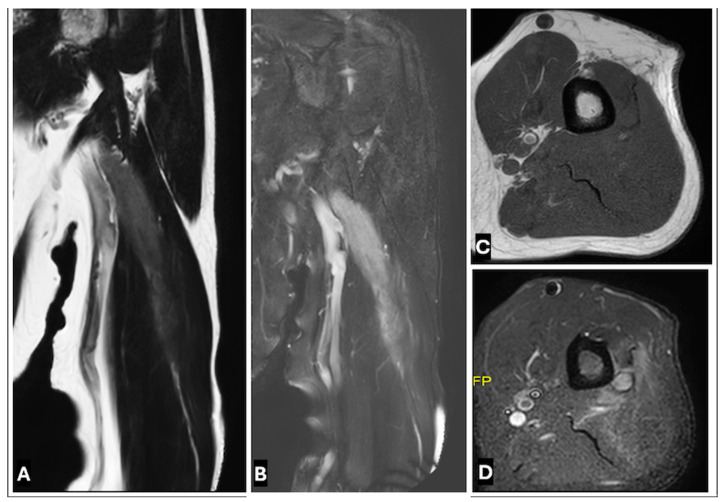
Intraneural lymphoma of radial nerve in a 38-year-old women (**A**–**D**). (**A**) T1-w coronal, (**B**) T2-w FS coronal, (**C**) T1-w axial, and (**D**) T2-w FS axial images showing lymphoma of radial nerve with diffuse nerve enlargement with abnormal T2-w high signal.

**Figure 21:**
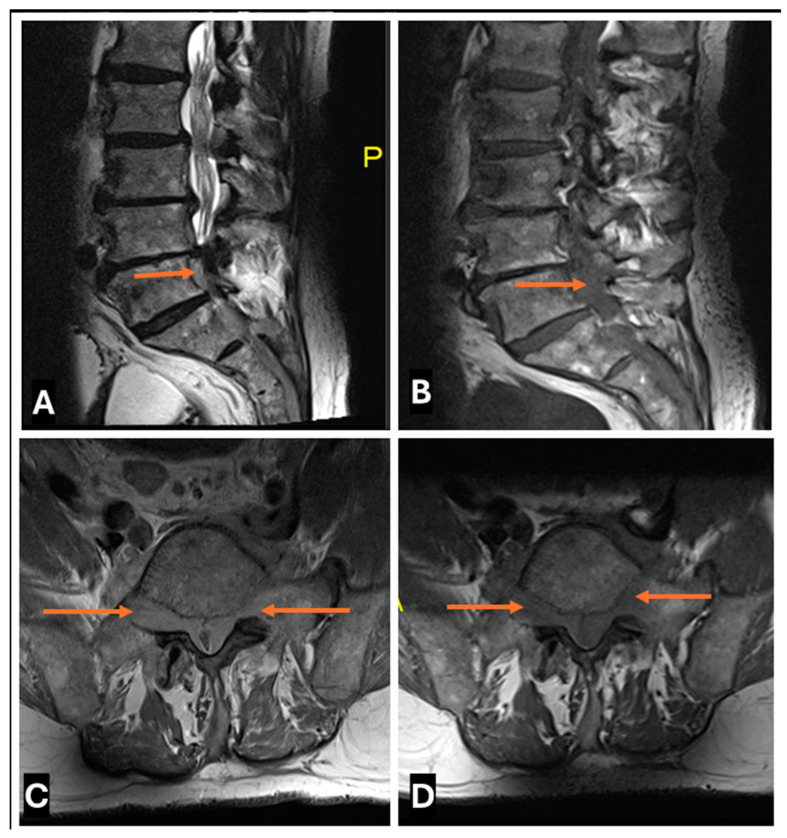
Neurolymphomatosis in a 45-year old male: (**A**–**D**) (**A**) T2-w Sagittal; (**B**) T1-w Sagittal; (**C**) T2-w Axial (**D**) T1-w Axial images showing Neurolymphomatosis as diffuse enlargement of multiple cauda equina nerve nerve roots (thin arrows) showing abnormal T2-w high signal.

**Figure 22:**
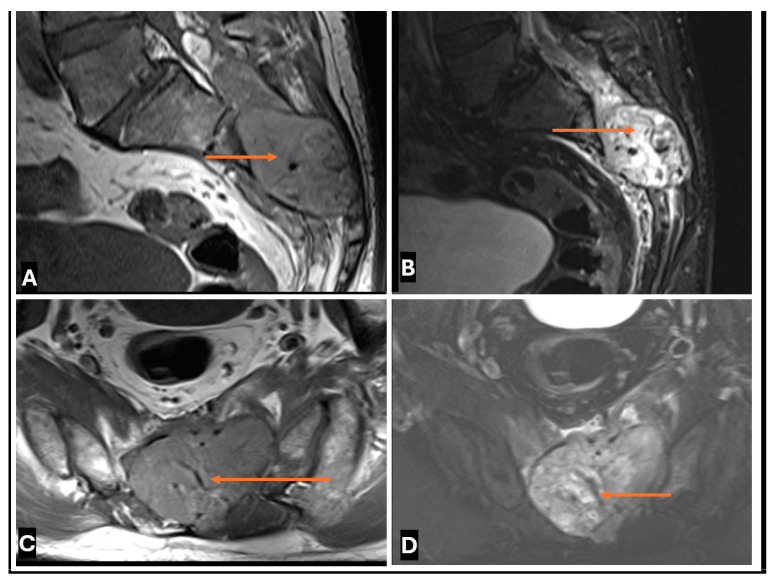
Benign intraneural hemangioblastoma in a 36-year old male. (**A**–**D**). (**A**) T1-w Coronal; (**B**) T2-w Sagittal; (**C**) T1-w axial and (**D**) T2-FS axial images showing intraneural hemangioblastoma (thin arrows) arising from a sacral nerve root within the dural sac with intermediate T1 signal and high T2 signal and presence of intralesional flow-voids.

**Figure 23:**
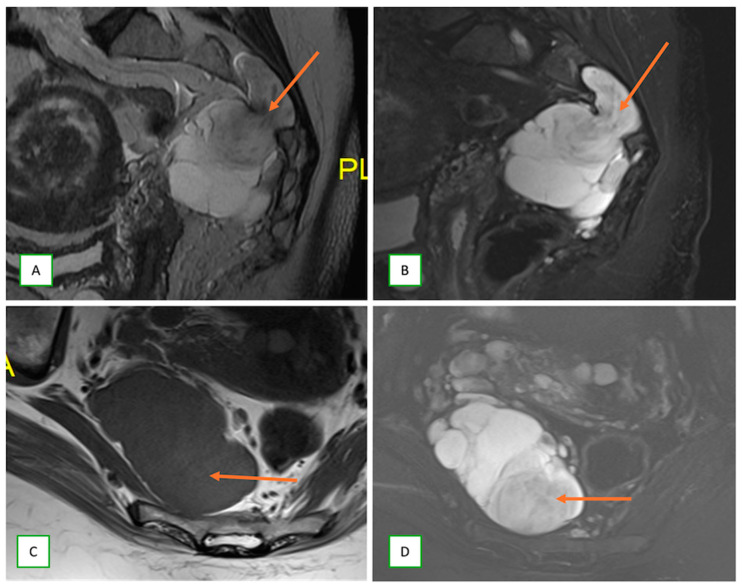
A hybrid tumor (neurofibroma–schwannoma complex) in a 42-year-old man. Sagittal T1 (**A**), sagittal fat-saturated T2 (**B**), axial T1 (**C**), and fat-saturated T2 (**D**) images showing a lesion of the right S3 nerve root (thin arrows) with mixed features of both schwannoma and neurofibroma.

**Figure 24:**
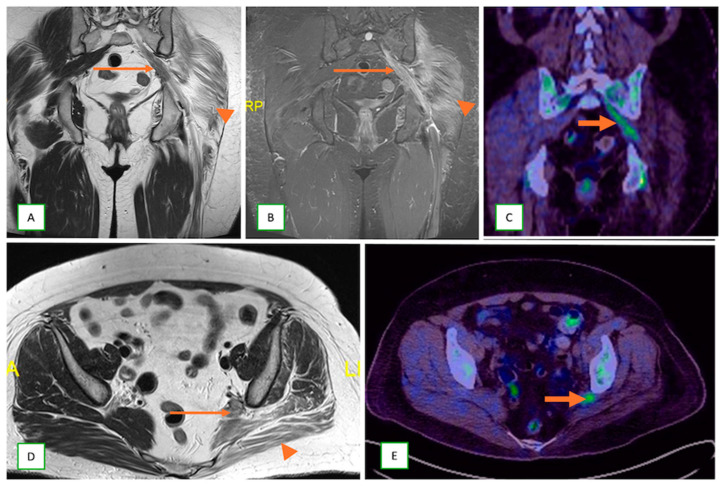
Endometrial carcinoma in a 35-year-old woman. Coronal T1 (**A**), coronal fat-saturated T2 (**B**), coronal fused PET-CT (**C**), axial T2 (**D**), and axial fused PET-CT (**E**) images showing endometriotic deposits in the left sciatic nerve (thin arrows) with FDG-avid activity demonstrated on FDG-PET fused images (thick arrows). Denervation edema and fatty atrophy are also seen in the left-sided gluteal muscles (arrowheads).

**Figure 25:**
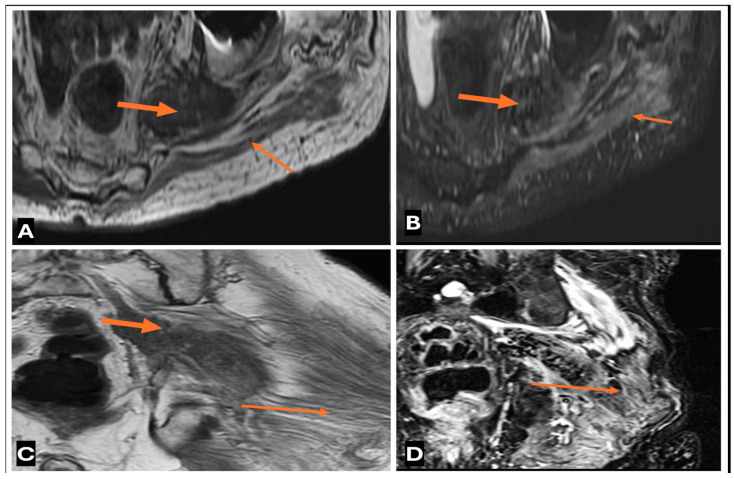
Metastatic deposits along the sciatic nerve from Prostate CA primary in a 55-year old male. (**A**–**D**) (**A**) T1-w Axial; (**B**) T2-w FS Axial; (**C**) T1-w Coronal (**D**) T2-w FS Coronal images showing Metastatic deposits (thick arrows) along the sciatic nerve from Prostate CA primary. Note the heterogenous metastatic deposit along the sacral nerve with resultant denervation edema (thin arrows) along the left sided gluteal muscles.

**Figure 26:**
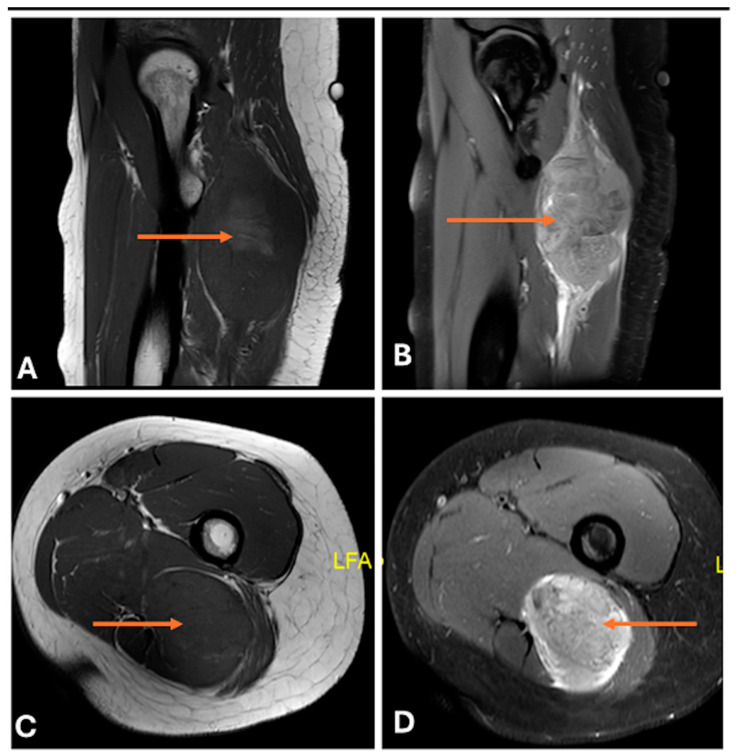
Primary Ewing’s sarcoma in the femoral nerve in a 28-year-old female. (**A**–**D**) (**A**) T1-w sagittal, (**B**) T2-w FS sagittal, (**C**) T1-w axial, and (**D**) T2-w FS axial images showing soft tissue Ewing’s sarcoma as a large, heterogeneous mass (thin arrows), with low to intermediate T1 signal and high T2 signal.

**Figure 27:**
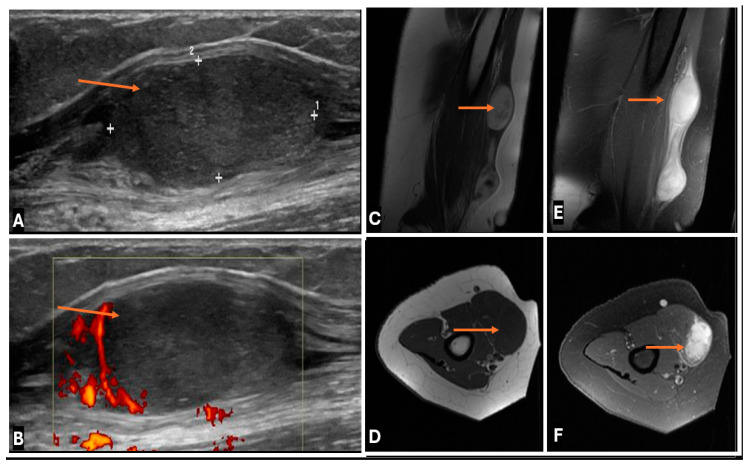
Primary Spindle cell sarcoma of the median nerve in a 32 year old female. (**A**–**F**) (**A**) Gray scale image (**B**) Color doppler image (**C**) T2-w Sagittal; (**B**) T2-w FS Sagittal; (**C**) T1-w Axial (**D**) T2-w FS Axial images showing Spindle cell sarcoma of the median nerve in the arm as relatively well circumscribed mass (thin arrows), with low to intermediate T1 signal and high T2 signal on MRI and appears hypoechoic on ultrasound with peripheral vascularity on colour doppler images.

**Figure 28:**
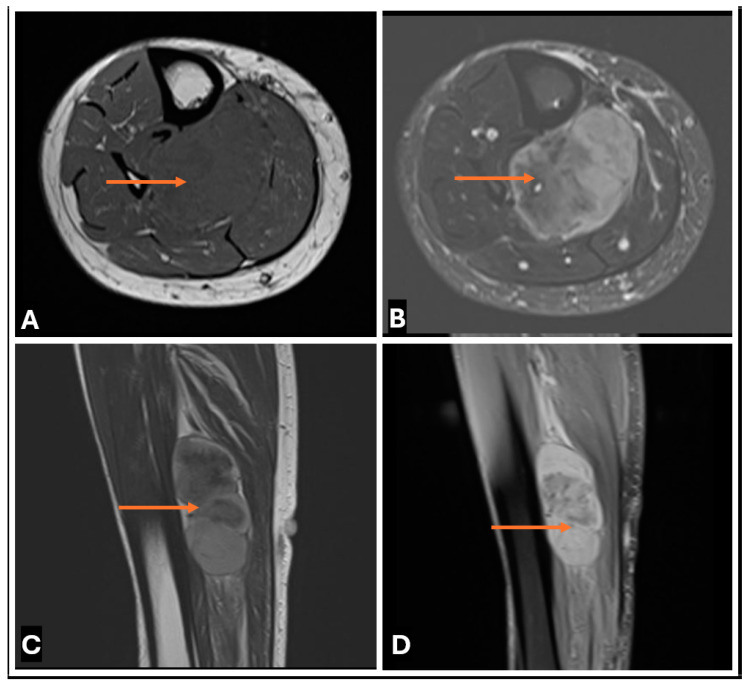
Primary Synovial sarcoma of the tibial nerve in a 30 year old female. (**A**–**D**) (**A**) T1-w Axial; (**B**) T2-w FS Axial; (**C**) T1-w Sagittal (**D**) T2-w FS Sagittal images showing Synovial sarcoma of the tibial nerve within the mid-lower leg as a lobulated heterogenous mass (thin arrows), with low to intermediate T1 signal and high T2 signal on MRI.

Finally, and most significantly, it is crucial to exclude other soft tissue tumors on imaging, which can mimic intraneural tumors. Several key differential diagnoses warrant consideration, including the following:

1. Dermal nerve sheath myxomas or neurothekeomas occur as small, painless nodules in young adults; they appear hypoechoic on USG and markedly hyperintense on T2-w images (Figure 14).

2. Lipofibromatous hamartoma (lipomatosis) of the nerve is characterized by fibro-fatty tissue proliferation within the epineurium, displaying a coaxial cable-like morphology on axial MR images and a spaghetti-like configuration on longitudinal MR images [29] (Figure 15).

3. Intraneural ganglion cysts usually occur near joints; are particularly common in the common peroneal nerve at the knee, owing to the intraarticular branch of the proximal tibiofibular joint; and appear as tubular, multiloculated cystic lesions following the course of the nerve on MRI [60,61] (Figure 16).

4. Intraneural perineurioma present as fusiform enlargements of the affected nerve with proximal and distal tapering and show avid enhancement on postcontrast MRI, often indistinguishable from other PNSTs [62] (Figure 17).

5. Benign triton tumors, or neuromuscular choristomas, are rare congenital lesions with a mix of neural and striated muscle elements, usually appearing as fusiform nerve enlargements with heterogeneous signals on MRI and often mimicking an aggressive lesion. They typically present in the first two decades (Figure 18).
6. Ganglioneuromas are rare, benign tumors of the sympathetic nervous system that are commonly identified incidentally and appear as well-defined, homogeneous masses, containing calcifications and showing mild to moderate enhancement on CT/MR in the posterior mediastinum, retroperitoneum, or adrenal gland (Figure 19).
7. Neurolymphomatosis describes intraneural spread of lymphoma and appears as diffuse enlargement of nerves, plexuses, spinal cord, and cauda equina. It shows more homogeneous enhancement than PNSTs and may be associated with lymphadenopathy or a known history of lymphoma [63] (Figure 20 and Figure 21).

8. Intraneural hemangioblastoma: Intraneural hemangioblastoma is a rare vascular malformation that occurs within a nerve and appears on MRI as an enlarged lobulated mass with internal flow voids and aggressive postcontrast enhancement [64] (Figure 22).
9. Traumatic neuromas: These neuromas occur at the site of a nerve injury or amputation and appear as a focal mass in continuity with the injured nerve, often with surrounding scarring [51].
10. Amyloidoma are rare and feature tumor-like amyloid deposition in peripheral nerves. They can mimic PNST but often show more diffuse nerve involvement [65].
11. Hybrid nerve sheath tumors, such as schwannoma–neurofibroma or schwannoma–perineurioma combinations, present with complex imaging features that can mimic more aggressive tumors; a definitive diagnosis necessitates immunohistochemistry and histopathological analysis (Figure 23).

12. Metastatic lesions: These include perineural spread of the tumor or metastasis to peripheral nerves by an adjacent or distant primary tumor. They are commonly observed with head and neck cancers, such as squamous cell carcinoma. They typically appear as linear thickening and enhancement extending along the course of the affected nerve on MRI with end muscle edema and appear FDG-avid on PET-CT [66] (Figure 24 and Figure 25).

13. Primary Sarcomas: Soft-tissue sarcomas are uncommon, accounting for just over 1% of adult malignancies. Synovial sarcoma, clear cell sarcoma, and epithelioid sarcomas are known to involve peripheral nerves. CT/MR reveals a large soft tissue mass with areas of necrosis or calcification and heterogeneous contrast enhancement. F-18 FDG uptake is useful for both tumor staging and treatment assessment (Figure 26, Figure 27 and Figure 28).

## 5. Multidisciplinary Team Approach in the Diagnosis and Treatment of Intraneural Tumors

Each patient receives an optimal individualized treatment plan according to the tumor biology, individual clinical scenarios, and evidence-based treatment protocols. The optimal patient management method is established via a decision-making process involving a multidisciplinary tumor (MDT) board composed of interventionist musculoskeletal radiologists, pathologists, surgeons, and oncologists (Table 3).

### 5.1. Role of Interventional Musculoskeletal Radiologists

Musculoskeletal (MSK) radiologists leverage their imaging proficiency to assist surgeons in meticulously planning and performing tumor resections, concurrently conducting image-guided biopsies to ensure accurate histopathological sampling, while safeguarding the parent nerve, as well as the adjacent nerves and structures [67,68]. Radiologists may also help guide surgeons by placing imaging markers to help them locate small or deep tumors [69,70]. Radiofrequency ablation or cryoablation of select cases of intraneural tumors may also be performed [71,72,73]. Preoperative embolization may be warranted for hyper-vascularized tumors, to mitigate intraoperative hemorrhage, while image-guided interventions such as nerve blocks or corticosteroid injections can be utilized for tumor-related pain management [74].

### 5.2. Role of Pathologist

Pathologists play a vital role in the analysis of biopsy and surgical samples to classify various tumor cell types, validate surgical resection margins, verify the final histological grades of tumors, forecast treatment outcomes, and direct therapeutic strategies. Additionally, they employ immunohistochemistry techniques to distinguish between different peripheral nerve sheath tumors, such as identifying S100 protein positivity in schwannomas and EMA positivity or negativity in neurofibromas and schwannomas, respectively [75,76]. They also analyze MDM2 FISH amplifications in atypical lipomatous or well-differentiated liposarcomas located near peripheral nerves, and perform NF1 mutation assessments when there is uncertainty regarding the diagnosis of MPNSTs [77]. They help to categorize and subcategorize neoplasms as benign or malignant tumors by assessing mitotic activity, necrosis, and cellular atypia, among other factors, so that researchers can correlate them with different subclasses of intraneural tumors, leading to further research and classification of intraneural tumors [57,78,79].

### 5.3. Role of Musculoskeletal Oncologists

Musculoskeletal oncologists conduct clinical evaluations, correlate patient history, and utilize imaging alongside physical tests for managing intraneural tumors [80]. Careful coordination and interpretation of imaging studies and biopsies is performed to reach a correct definitive diagnosis [81]. They collaborate with several other clinicians to determine MPNST primary staging [82] and develop an individualized treatment plan according to the tumor biology, site specificity, and patient background [83]. The medical treatment of MPNSTs and other malignancies is usually chemotherapy [84] with long-term follow-up to prevent tumor recurrence and side effects [85].

### 5.4. Role of Onco-Surgeon

The surgeon assesses surgical viability based on the tumor’s imaging features and proximity to vital structures [54], aiming for complete excision while preserving neural function, aided by intraoperative monitoring with neurophysiologists [86,87]. In cases where nerve resection is planned, extensive nerve grafts or reconstructions are performed in succession, to maintain nerve continuity [88]. Whenever possible, limb-salvage procedures are prioritized to maintain patient mobility [89], although amputations may be required for larger malignant tumors [90].

### 5.5. Role of Radiation Oncologists

Radiation oncologists help develop a comprehensive radiotherapy plan that includes the radiation dosage, fractionation, and administration methods [91]. Neoadjuvant radiotherapy can effectively decrease tumor size prior to surgery, facilitating easier excision [92], whereas postoperative radiation is crucial for minimizing local recurrence in patients with positive resection margins [93]. Definitive radiation therapy is typically designated for tumors that are unresectable or for patients unsuitable for surgery [94,95], with additional techniques such as stereotactic radiosurgery or brachytherapy considered for specific cases [96,97].

## 6. Biopsy and Treatment Considerations

A biopsy is essential when imaging findings are atypical or equivocal or have concerning imaging findings for malignancy; a definitive diagnosis will affect clinical management, and preoperative histological confirmation is needed to aid treatment planning [90]. However, “do not touch” lesions, such as typical schwannomas in noncritical areas, small asymptomatic neurofibromas, and lesions highly suspicious for low-grade MPNSTs in NF1 patients (for whom wide excision is the standard of care), generally do not require biopsy.

Imaging guidance via ultrasound or CT is essential for biopsy procedures, because it facilitates precise tissue targeting, avoids neurovascular structures, and enhances the accuracy of diagnostics by ensuring that adequate samples are obtained from the most clinically relevant regions of the tumor.

The choice of treatment depends upon the tumor characteristics [benign or malignant, size, and location], patient factors [age, overall health, and surgical fitness], and anticipated functional outcomes. Benign tumors that are small and asymptomatic are generally observed through a “watchful waiting” strategy involving regular clinical and imaging evaluations and short-interval clinical and imaging assessments [98]. Surgical excision remains the primary treatment for symptomatic or enlarging benign lesions and localized malignancies [86], aiming for complete tumor removal, while safeguarding parent nerve integrity, particularly in schwannomas, with favorable outcomes and low recurrence rates post-enucleation surgery [99].

Resection with interfascicular dissection [100] is effective for single neurofibromas, whereas extensive debulking may be necessary for NF1-associated plexiform neurofibromas because of their infiltrative nature. Surgical interventions often involve intraoperative nerve stimulation to protect functional nerve fascicles, with microsurgical techniques employed to mitigate nerve damage [101,102].

Malignant peripheral nerve sheath tumors necessitate aggressive surgical resection with negative margins [101], aiming for negative resection margins and even requiring sacrifice of the involved nerve at times. Surgical interventions may include nerve reconstruction techniques or amputation for severe cases.

Postoperative radiation therapy is recommended to lower the risk of local recurrence, particularly in patients with positive or narrow resection margins [103]. Primary radiotherapy is reserved for unresectable tumors [94]. Stereotactic radiosurgery can be performed for small, well-defined tumors in critical locations [96,104].

Ongoing clinical trials are exploring innovative therapies, including targeted immune treatments (e.g., MEK inhibitors such as selumetinib for the treatment of NF1-related plexiform neurofibromas) [105,106]. Multimodal approaches, including a combination of surgery, radiation, and systemic treatments for complex cases, may also be considered [81,107].

Adjuvant chemotherapy remains controversial but may be utilized in high-risk patients [84]. Preoperative neoadjuvant radiation or chemotherapy may help reduce the tumor size and increase its resectability. Palliative care focuses on symptom relief and clinical trials are exploring novel therapies for aggressive tumors [95,108] (Table 4).

## 7. Postoperative Imaging

Postoperative imaging is critical for evaluating postsurgical outcomes, screening for recurrence, evaluating neurological deficits following large-volume resection, and determining the further course of treatment. The excellent soft tissue resolution capability of MR helps in detailed visualization of the postsurgical tumor bed and surrounding structures, as well as detection of any residual or recurrent tumors [109]. Postoperative MR images are often difficult to interpret, requiring not only knowledge of the clinical presentation and surgical techniques but also comprehension of the expected postsurgical changes.

Several key imaging features and techniques can help with postoperative imaging assessment. Residual or recurrent tumors often show abnormal signal intensity, similar to that of the original lesion [110]. Residual or recurrent tumors typically appear hyperintense on T2 and STIR images and tend to persist or progress [111], whereas postoperative edema usually resolves over time. Residual or recurrent tumors often show enhancement similar to that of the original tumor [112]. Fat-suppressed sequences are better at visualizing enhancing tissue in postoperative beds [40]. Nodular or mass-like areas with irregular margins are more suspicious for residual/recurrent tumors [113]. It is important to be cautious of thin linear enhancement along the surgical tract, as this may result from normal postoperative changes. DCE imaging can help differentiate tumor recurrence from posttreatment changes based on enhancement kinetics [40]. Residual or recurrent tumors often show restricted diffusion [114]. A comparison of apparent diffusion coefficient (ADC) values with those of preoperative tumors can also be helpful. Comparison of postoperative findings with preoperative images can identify new or changed areas of abnormality [34]. Changes in the tumor volume of any suspicious area compared with previous scans can help detect any interval growth; however, caution must be exercised, as early postoperative scans (within 48–72 h) tend to overestimate the residual tumor volume [115]. Although not routinely used, PET/CT can differentiate tumor recurrence from radiation necrosis or postsurgical changes [116]. In cases where MRI alone is inconclusive, PET/MRI can provide additional metabolic information to detect recurrence [117]. Abnormal thickening or enhancement along the course of the affected nerve may indicate the perineural spread of the tumor [118]. Notably, muscle denervation changes can mimic tumor recurrence. Identifying characteristic patterns of muscle signal changes during denervation can aid in diagnosis [4]. Regular follow-up imaging is crucial for detecting subtle changes that may indicate recurrence [22]. In cases of uncertainty, short-interval follow-up imaging (three months, six months, one year, and then yearly follow-up) or biopsy may be necessary.

## 8. Newer Imaging Advances

Advances in technology have significantly enhanced the diagnosis and treatment of intraneural tumors. Radiomics helps to extract and analyze quantitative features from medical images to distinguish benign from malignant intraneural tumors [119,120,121]. Innovative artificial intelligence (AI) algorithms utilizing deep learning (DL) and complex neural networks (CNNs) are being developed to identify, characterize, and forecast malignant transformations in peripheral nerve sheath tumors [122]. To improve INT tissue characterization, novel MRI techniques such as CEST imaging, MRE, QSM, and PET/MRI hybrid imaging are being studied. PET tracers targeting somatostatin receptors or specific molecular markers may improve INT functional imaging, help distinguish tumor types, and assess treatment response [123,124]. Diagnostic imaging and targeted therapy (“theranostics”) may inspire MPNST treatment strategies [125]. In some tumors, radiolabeled somatostatin analogues can be used for imaging and targeted radiotherapy. Since liquid biopsy is non-invasive and identifies circulating tumor DNA or other biomarkers in blood samples, it could change MPNST imaging and monitoring diagnostics [126]. Complex surgery can be planned using patient-specific 3D-printed models.

## 9. Conclusions

MRI is the gold standard for assessing intraneural malignancies, with basic as well as advanced MR sequences providing comprehensive insights into tumor morphology and helping guide biopsy and surgical planning. This review illustrates how systematic and thorough evaluation of key imaging features can help narrow the differential diagnosis between benign and malignant intraneural tumors, aid in clinical decision-making by cross-referencing imaging and histopathological findings, help precise biopsy targeting and presurgical planning, detect malignant transformation in benign lesions, and assist with posttreatment surveillance.

## Figures and Tables

**Table 1:** MRI sequences and rationale.

MRI Sequence	Rationale
**Axial T1-weighted 3.0-mm**	Defines normal nerve anatomy; loss of signal from fat suggests nerve involvement.
**Axial T2-weighted with fat saturation**	Distinguishes tumors from fat and highlights intraneural cystic/oedematous components.
**Axial T2-weighted STIR**	Excellent for detecting nerve inflammation and cystic lesions.
**Axial Diffusion-weighted imaging (DWI)**	Detects highly cellular tumors; ADC values help distinguish benign vs. malignant tumors.
**Coronal/Sagittal T2-weighted FS or STIR**	Assesses nerve root and plexus involvement; highlights tumor–nerve relationship and extension along fascicles.
**MR Neurography (MRN)**	High-resolution nerve imaging (3D STIR/SPACE) to delineate fascicular architecture and perineural tissues.
**Sagittal/Coronal Diffusion Tensor Imaging (DTI)**	Quantifies nerve integrity; evaluates directionality of nerve fibers to identify disruption by the tumor.
**Axial T1-weighted with Gadolinium FS**	Enhances tumoral components and tumor delineation from surrounding tissues.
**Sagittal/Coronal T1-weighted with Gadolinium FS**	Assesses the extent of nerve involvement, especially post-treatment follow-up, to detect residual or recurrent tumor.
**Dynamic Contrast-Enhanced (DCE) imaging**	Evaluates perfusion characteristics of the tumor to differentiate benign from malignant based on enhancement kinetics.

**Table 2:** MRI characteristics and differentiation of benign peripheral nerve sheath tumors (PNSTs) from malignant peripheral nerve sheath tumors (MPNSTs).

Feature	Benign PNSTs (BPNSTs)	Malignant PNSTs (MPNSTs)
**Size**	Generally smaller (<5 cm).	Typically larger (>5 cm).
**Margins**	Well-defined, smooth margins.	Ill-defined, irregular margins.
**Growth Rate**	Slow-growing, stable over time.	Rapid growth or change in size over time.
**T1-w Imaging**	Isointense to slightly hypointense relative to muscle.	Isointense to hypointense relative to muscle, often heterogeneous.
**T2-w Imaging**	Hyperintense with possible “target sign” (central low, peripheral high signal).	Hyperintense, often heterogeneous without a clear “target sign”.
**Enhancement Pattern**	Homogeneous or mildly heterogeneous enhancement.	Markedly heterogeneous enhancement, often irregular or nodular.
**Cystic Degeneration**	Common, especially in schwannomas.	May be present but often associated with necrosis.
**Peritumoral Oedema**	Usually absent or minimal.	Frequently present with surrounding soft tissue oedema.
**Invasion of Adjacent Structures**	Absent; typically does not invade surrounding tissues.	Present; may invade adjacent muscles, bones, or neurovascular structures.
**Bone Involvement**	Absent; no bone destruction.	May cause bone erosion or destruction if adjacent to bony structures.
**Nerve Enlargement**	Fusiform enlargement, often uniform.	Irregular nerve enlargement, may show abrupt transitions.
**Pain**	Often less painful; pain is not a predominant feature.	Frequently associated with pain and neurological deficits.
**Tinel’s Sign**	May be positive, especially in schwannomas.	Can be positive, but often with more severe symptoms.
**Clinical Context**	Commonly associated with neurofibromatosis type 1 (NF1) or sporadic.	Often associated with neurofibromatosis type 1 (NF1) or history of prior radiation.

**Table 3:** Multidisciplinary tumor management—key information required at the MDT.

Specialty	Key Information Required
**Interventional Radiologist (Pre-biopsy)**	Tumor location, size, proximity to vital structures (e.g., vessels, nerves). Pathology suspicion (benign vs. malignant). Safe access route.
**Surgeon (Surgical Planning)**	Extent of tumor involvement with surrounding tissues. Tumor relationship with critical structures (e.g., vessels, nerves). Potential for nerve sparing or resection margins.
**Radiation Oncologist (Radiation Planning for MPNST)**	Tumor size and volume for treatment field planning. Proximity to radio-sensitive organs. Clear delineation of tumor margins. Presence of residual or recurrent disease.
**Radiologist (Post-treatment Imaging)**	Presence of residual tumor or recurrence. Post-surgical changes. Enhancing versus non-enhancing tissue. Nerve regeneration or tumor regrowth.
**Radiologist (Malignant Transformation of Benign Tumor)**	Size, growth rate, and signal changes compared to previous studies. Increased enhancement, heterogeneity, or invasion into adjacent structures. Presence of new necrosis or perineural invasion.

**Table 4:** Radiology reporting tips and recommendations for MR reporting of INTs.

**Diagnosis:**	Employ a standardized reporting framework to ensure thoroughness and uniformity. Examine for direct continuity with neural structures or along standard nerve distributions. Identify distinctive MR imaging characteristics of INTs, including split-fat, target, and fascicular signs. Assess the INT in three dimensions and record any size variations from prior examinations. Ill-defined margins, heterogeneous signals, and peritumoral edema are all indicators of potential malignancy. Integrating diffusion-weighted imaging (DWI) and apparent diffusion coefficient (ADC) maps can enhance diagnostic precision. Explore advanced imaging methodologies like MR neurography or diffusion tensor imaging (DTI) for superior nerve visualization.
**Intervention:**	Always choose the safest approach during image-guided biopsies, avoiding disruption of intact nerve fascicles. Utilize high-resolution ultrasound for real-time monitoring during biopsy procedures.
**Treatment Planning:**	Accurately depict the tumor’s relationship to critical neighbouring structures, such as major blood vessels and muscles. Provide precise anatomical localization to facilitate surgical strategy. Evaluate the possibility of nerve-sparing resection based on imaging data. Investigate local invasion and distant metastasis in suspected malignant cases.
**Management**	Suggest appropriate follow-up schedules based on the tumor’s classification and grade. For neurofibromatosis patients, recommend whole-body MRI screening when warranted. In suspected malignancy cases, PET/CT or PET/MRI for metabolic evaluation is advised.
**Post-operative Care**	Understand normal post-operative imaging appearances to prevent misinterpretation. Implement fat-suppressed sequences to enhance visualization of post-operative enhancing tissues.
**General Recommendation**	Ensure clear communication with referring clinicians about significant or unexpected findings. Engage in multidisciplinary tumor boards to gain and share imaging insights and clinical knowledge. Stay well-updated with the current WHO classification of peripheral nerve sheath tumors. Knowledge of genetic syndromes associated with these tumors (NF1, NF2, schwannomatosis) aids diagnosis. Recommend genetic counselling for patients with multiple tumors or younger patients with solitary tumors.

## Data Availability

Data can be shared on request.
